# Decacarbon­yl[μ_4_-(ethane-1,2-diyl­dinitrilo)­tetra­kis­(methane­thiol­ato)]bis(triphenyl­phosphane)tetra­iron(2 *Fe*—*Fe*)

**DOI:** 10.1107/S160053681105584X

**Published:** 2012-01-07

**Authors:** Wei-Ming Gao, Jia-Ming Li

**Affiliations:** a Laboratory of Chemical Genomics, School of Chemical Biology and Biotechnology, Graduate School of Peking University, Shenzhen, Guangdong 518055, People’s Republic of China; b College of Chemistry and Chemical Engineering, Qinzhou University, Qinzhou, Guangxi 535000, People’s Republic of China

## Abstract

In the title compound, [Fe_4_(C_6_H_12_N_2_S_4_)(C_18_H_15_P)_2_(CO)_10_], the unit cell contains one mol­ecule, which exhibits a crystallographically imposed center of symmetry. The independent Fe_2_S_2_ fragment [Fe—Fe = 2.527 (1) Å] is in a butterfly conformation, and each Fe atom displays a pseudo-square-pyramidal coordination geometry. The phosphane group occupies an apical position [Fe—P = 2.2670 (14) Å]. In the crystal, weak inter­molecular C—H⋯O hydrogen bonds link the mol­ecules into chains along [110].

## Related literature

For background to macrocyclic complexes containing butterfly [Fe_2_S_2_] clusters, see: Gloaguen & Rauchfuss (2009 ▶); Yin *et al.* (2011 ▶); Zhao *et al.* (2009 ▶). For related structures containing butterfly [Fe_2_S_2_] clusters, see: Liu *et al.* (2011 ▶); Liu & Yin (2011 ▶); Song *et al.* (2011 ▶); Gao *et al.* (2011 ▶). For details of the synthesis, see: Gao *et al.* (2011 ▶).
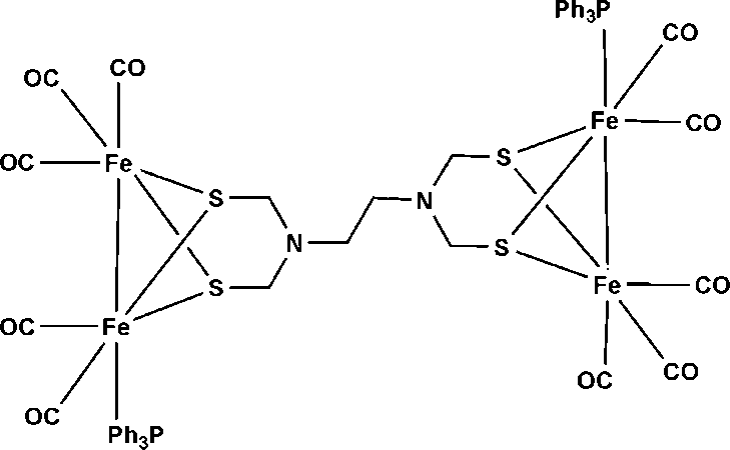


## Experimental

### 

#### Crystal data

[Fe_4_(C_6_H_12_N_2_S_4_)(C_18_H_15_P)_2_(CO)_10_]
*M* *_r_* = 1268.46
Triclinic, 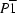

*a* = 10.854 (2) Å
*b* = 11.995 (2) Å
*c* = 12.202 (3) Å
α = 63.257 (3)°
β = 71.881 (3)°
γ = 74.736 (3)°
*V* = 1334.0 (5) Å^3^
*Z* = 1
Mo *K*α radiation
μ = 1.34 mm^−1^
*T* = 296 K
0.30 × 0.20 × 0.15 mm

#### Data collection

Bruker SMART CCD area-detector diffractometer
Absorption correction: multi-scan (*SADABS*; Sheldrick, 1996 ▶) *T*_min_ = 0.732, *T*_max_ = 0.818
7640 measured reflections
5124 independent reflections
3098 reflections with *I* > 2σ(*I*)
*R*_int_ = 0.072

#### Refinement

*R*[*F* ^2^ > 2σ(*F* ^2^)] = 0.055
*wR*(*F* ^2^) = 0.090
*S* = 1.01
5124 reflections
334 parameters
H-atom parameters constrained
Δρ_max_ = 0.56 e Å^−3^
Δρ_min_ = −0.61 e Å^−3^

### 

Data collection: *SMART* (Bruker, 2007 ▶); cell refinement: *SAINT* (Bruker, 2007 ▶); data reduction: *SAINT*; program(s) used to solve structure: *SHELXS97* (Sheldrick, 2008 ▶); program(s) used to refine structure: *SHELXL97* (Sheldrick, 2008 ▶); molecular graphics: *SHELXTL* (Sheldrick, 2008 ▶); software used to prepare material for publication: *SHELXTL*.

## Supplementary Material

Crystal structure: contains datablock(s) I, global. DOI: 10.1107/S160053681105584X/cv5221sup1.cif

Structure factors: contains datablock(s) I. DOI: 10.1107/S160053681105584X/cv5221Isup2.hkl

Additional supplementary materials:  crystallographic information; 3D view; checkCIF report

## Figures and Tables

**Table 1:** Hydrogen-bond geometry (Å, °)

*D*—H⋯*A*	*D*—H	H⋯*A*	*D*⋯*A*	*D*—H⋯*A*
C20—H20*A*⋯O5^i^	0.93	2.39	3.182 (7)	143

Symmetry code: (i) 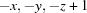.

**Fig. 1.**
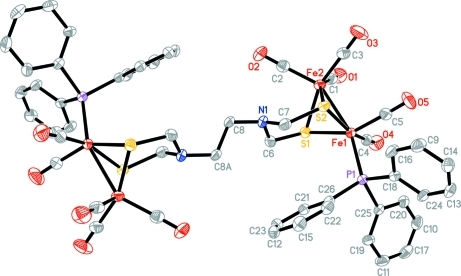
The molecular structure of the title compound, with displacement ellipsoids at the 30% probability level [symmetry code: (A) 1-x, 1-y, 1-z]. H atoms have been omitted for clarity.

**Fig. 2.**
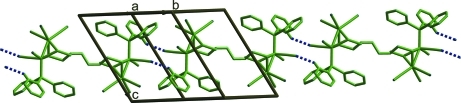
Part of the zigzag infinite chain linked via hydrogen bonds (dashed lines) in [110] direction. H atoms have been omitted for clarity, except for those involved in hydrogen-bonded interactions.

## supplementary crystallographic information

### Comment

Macrocyclic complexes containing butterfly [Fe_2_S_2_] clusters have aroused considerable attention due to their unique structures and interesting phyical and chemical properties (Gloaguen & Rauchfuss, 2009; Yin *et al.*, 2011; Zhao *et al.*, 2009). In recent years, Liu and co-workers reported a series of macrocyclic complexes (Liu, Xiao *et al.*, 2011; Liu & Yin, 2011) with the structure of active site of [FeFe]-hydrogenases. Following the above consideration and ongoing our works in this field (Gao *et al.*, 2011), we report here a dimer structure of the title compound (I) - a new structure model of Fe_2_S_2_ cluster.

The title molecule (Fig. 1) lies across a crystallographic inversion centre which is situated at the midpoint of the C8–C8A (1.552 (8) Å, symmetry code: (A) 1-x, 1-y, 1-z) bond. The independent Fe_2_S_2_ fragment [Fe—Fe 2.527 (1) Å] is in a butterfly conformation, and each Fe atom displays pseudo square-pyramidal coordination geometry. The phosphane group occupies an apical position [Fe—P 2.2670 (14) Å], while the (thiomethyl)ethane-1,2-diamine group on the bridging N atom is in an equatorial position and takes a zigzag form. Complex (I) contains two fused six-membered rings, in which one six-membered ring (N1C7S2Fe2S1C6) has a chair conformation and the other six-membered ring (N1C7S2Fe1S1C6) has a boat conformation. The substituent attached to the bridgehead N1 lies in an equatorial position and the unpaired electrons of nitrogen lie in an axial position which is consistent with corresponding diiron azadithiolate complexes (Gao *et al.*, 2011). The sum of the C–N–C angles around nitrogen is 342.9 °, which means there is no π–π conjugation between the substituent group and the p-orbital of nitrogen.

In the crystal structure, intermolecular C—H···O hydrogen bonds (Table 1) link the molecules into infinite chains along the [110] direction (Fig. 2).

### Experimental

A solution of [{Fe_2_(CO)_5_µ-(SCH_2_)_2_NCH_2_CH_2_Nµ-(SCH_2_)_2_Fe_2_(CO)_5_}(PPh_3_)_2_] (0.4 g, 0.5 mmol) and Me_3_NO.2H_2_O (0.111 g, 1 mmol) dissolved in MeCN (40 mL) was stirred for 5 to 10 min at room temperature. Then, a solution of PPh_3_ (0.524 g, 1 mmol), dissolved in CH_2_Cl_2_ (2 mL) was added. After 1 h, the solvent was evaporated, and the crude product was purified by chromatography on silica gel with CH_2_Cl_2_/hexane (1/2 v/v) as the eluent to give the crystals suitable for X-ray diffraction study. Elemental analysis (%) calcd for C_52_H_42_Fe_4_N_2_O_10_P_2_S_4_: C, 49.24; H, 3.34; N, 2.21. Found: C, 49.25; H, 3.39; N, 2.19.

### Refinement

All H atoms were placed in idealized positions (C—H = 0.93–0.97 Å) and refined as riding atoms, with *U*_iso_(H) = 1.2*U*_eq_(C).

### Crystal data

**Table:**

[Fe_4_(C_6_H_12_N_2_S_4_)(C_18_H_15_P)_2_(CO)_10_]	*Z* = 1
*M**_r_* = 1268.46	*F*(000) = 646
Triclinic, *P*1	*D*_x_ = 1.579 Mg m^−^^3^
Hall symbol: -P 1	Mo *K*α radiation, λ = 0.71073 Å
*a* = 10.854 (2) Å	Cell parameters from 1603 reflections
*b* = 11.995 (2) Å	θ = 2.4–22.1°
*c* = 12.202 (3) Å	µ = 1.34 mm^−^^1^
α = 63.257 (3)°	*T* = 296 K
β = 71.881 (3)°	Block, red
γ = 74.736 (3)°	0.30 × 0.20 × 0.15 mm
*V* = 1334.0 (5) Å^3^	

### Data collection

**Table:**

Bruker SMART CCD area-detector diffractometer	5124 independent reflections
Radiation source: fine-focus sealed tube	3098 reflections with *I* > 2σ(*I*)
graphite	*R*_int_ = 0.072
phi and ω scans	θ_max_ = 26.1°, θ_min_ = 1.9°
Absorption correction: multi-scan (*SADABS*; Sheldrick, 1996)	*h* = −13→11
*T*_min_ = 0.732, *T*_max_ = 0.818	*k* = −14→13
7640 measured reflections	*l* = −14→15

### Refinement

**Table:**

Refinement on *F*^2^	Primary atom site location: structure-invariant direct methods
Least-squares matrix: full	Secondary atom site location: difference Fourier map
*R*[*F*^2^ > 2σ(*F*^2^)] = 0.055	Hydrogen site location: inferred from neighbouring sites
*wR*(*F*^2^) = 0.090	H-atom parameters constrained
*S* = 1.01	*w* = 1/[σ^2^(*F*_o_^2^) + (0.005*P*)^2^] where *P* = (*F*_o_^2^ + 2*F*_c_^2^)/3
5124 reflections	(Δ/σ)_max_ < 0.001
334 parameters	Δρ_max_ = 0.56 e Å^−^^3^
0 restraints	Δρ_min_ = −0.61 e Å^−^^3^

### Special details

**Table:**

Geometry. All esds (except the esd in the dihedral angle between two l.s. planes) are estimated using the full covariance matrix. The cell esds are taken into account individually in the estimation of esds in distances, angles and torsion angles; correlations between esds in cell parameters are only used when they are defined by crystal symmetry. An approximate (isotropic) treatment of cell esds is used for estimating esds involving l.s. planes.
Refinement. Refinement of F^2^ against ALL reflections. The weighted R-factor wR and goodness of fit S are based on F^2^, conventional R-factors R are based on F, with F set to zero for negative F^2^. The threshold expression of F^2^ > 2sigma(F^2^) is used only for calculating R-factors(gt) etc. and is not relevant to the choice of reflections for refinement. R-factors based on F^2^ are statistically about twice as large as those based on F, and R- factors based on ALL data will be even larger.

### Fractional atomic coordinates and isotropic or equivalent isotropic displacement parameters (Å^2^)

**Table:**

	*x*	*y*	*z*	*U*_iso_*/*U*_eq_	
Fe1	0.15320 (7)	0.23740 (6)	0.47610 (6)	0.0398 (2)	
Fe2	0.30658 (7)	0.34428 (7)	0.26923 (6)	0.0454 (2)	
P1	0.08582 (12)	0.15121 (11)	0.68873 (11)	0.0381 (3)	
S2	0.37365 (12)	0.20598 (11)	0.44901 (11)	0.0431 (3)	
S1	0.17511 (12)	0.44442 (11)	0.39346 (11)	0.0456 (3)	
C26	0.1553 (4)	0.2004 (5)	0.7753 (4)	0.0370 (12)	
C25	−0.0910 (4)	0.1748 (4)	0.7554 (4)	0.0385 (12)	
N1	0.4172 (4)	0.4284 (4)	0.4331 (3)	0.0444 (11)	
C24	0.0359 (5)	−0.1018 (5)	0.8356 (4)	0.0471 (13)	
H24A	−0.0485	−0.0687	0.8661	0.057*	
C23	0.1836 (5)	0.3697 (5)	0.8167 (4)	0.0488 (14)	
H23A	0.1648	0.4544	0.8036	0.059*	
O4	−0.0977 (4)	0.3248 (4)	0.4041 (3)	0.0736 (12)	
O2	0.5107 (4)	0.5091 (4)	0.1278 (3)	0.0767 (13)	
C22	0.2369 (5)	0.1175 (5)	0.8561 (4)	0.0501 (14)	
H22A	0.2553	0.0326	0.8701	0.060*	
C8	0.5101 (5)	0.4944 (5)	0.4367 (4)	0.0556 (15)	
H8A	0.5050	0.5787	0.3706	0.067*	
H8B	0.5978	0.4510	0.4177	0.067*	
C21	0.1301 (4)	0.3260 (5)	0.7571 (4)	0.0445 (13)	
H21A	0.0757	0.3823	0.7035	0.053*	
C7	0.4326 (5)	0.2940 (5)	0.5059 (4)	0.0510 (14)	
H7A	0.3858	0.2775	0.5924	0.061*	
H7B	0.5247	0.2633	0.5058	0.061*	
C5	0.1659 (5)	0.0983 (5)	0.4589 (4)	0.0543 (15)	
C20	−0.1759 (5)	0.1481 (4)	0.7106 (4)	0.0519 (14)	
H20A	−0.1429	0.1233	0.6436	0.062*	
O3	0.4167 (4)	0.1517 (4)	0.1686 (4)	0.0926 (14)	
C6	0.2828 (5)	0.4894 (5)	0.4487 (4)	0.0541 (15)	
H6A	0.2823	0.5799	0.4046	0.065*	
H6B	0.2473	0.4714	0.5374	0.065*	
C19	−0.1426 (5)	0.2119 (4)	0.8561 (4)	0.0475 (13)	
H19A	−0.0870	0.2290	0.8894	0.057*	
C18	0.1256 (5)	−0.0211 (4)	0.7533 (4)	0.0408 (12)	
C3	0.3731 (5)	0.2276 (5)	0.2085 (5)	0.0573 (15)	
C17	−0.3569 (5)	0.1958 (5)	0.8585 (5)	0.0648 (16)	
H17A	−0.4467	0.2036	0.8924	0.078*	
C16	0.2516 (5)	−0.0765 (5)	0.7143 (4)	0.0537 (15)	
H16A	0.3153	−0.0254	0.6604	0.064*	
C2	0.4311 (5)	0.4440 (5)	0.1847 (4)	0.0544 (15)	
C4	0.0004 (6)	0.2890 (5)	0.4372 (5)	0.0513 (14)	
C15	0.2905 (5)	0.1613 (5)	0.9155 (4)	0.0548 (15)	
H15A	0.3445	0.1054	0.9697	0.066*	
C14	0.1934 (6)	−0.2823 (5)	0.8335 (5)	0.0593 (16)	
H14A	0.2159	−0.3694	0.8606	0.071*	
C13	0.0692 (6)	−0.2307 (5)	0.8735 (5)	0.0600 (16)	
H13A	0.0064	−0.2830	0.9270	0.072*	
C12	0.2649 (5)	0.2874 (6)	0.8954 (5)	0.0560 (16)	
H12A	0.3025	0.3164	0.9348	0.067*	
C11	−0.2760 (5)	0.2232 (5)	0.9060 (5)	0.0609 (16)	
H11A	−0.3105	0.2494	0.9720	0.073*	
C10	−0.3078 (5)	0.1569 (5)	0.7613 (5)	0.0584 (15)	
H10A	−0.3634	0.1367	0.7304	0.070*	
C1	0.1951 (5)	0.4157 (6)	0.1694 (5)	0.0597 (16)	
C9	0.2834 (5)	−0.2050 (5)	0.7537 (5)	0.0616 (16)	
H9A	0.3680	−0.2394	0.7253	0.074*	
O1	0.1203 (4)	0.4602 (4)	0.1054 (3)	0.0830 (13)	
O5	0.1750 (4)	0.0079 (4)	0.4432 (4)	0.0970 (15)	

### Atomic displacement parameters (Å^2^)

**Table:**

	*U*^11^	*U*^22^	*U*^33^	*U*^12^	*U*^13^	*U*^23^
Fe1	0.0444 (5)	0.0435 (5)	0.0331 (4)	−0.0135 (4)	−0.0038 (4)	−0.0161 (4)
Fe2	0.0527 (5)	0.0477 (5)	0.0316 (4)	−0.0118 (4)	−0.0030 (4)	−0.0141 (4)
P1	0.0382 (8)	0.0400 (8)	0.0348 (7)	−0.0102 (6)	−0.0030 (6)	−0.0147 (6)
S2	0.0446 (8)	0.0442 (8)	0.0371 (7)	−0.0105 (6)	−0.0037 (6)	−0.0147 (6)
S1	0.0502 (9)	0.0410 (8)	0.0427 (8)	−0.0092 (6)	−0.0071 (7)	−0.0149 (6)
C26	0.035 (3)	0.043 (3)	0.027 (3)	−0.016 (2)	0.002 (2)	−0.010 (2)
C25	0.035 (3)	0.038 (3)	0.037 (3)	−0.009 (2)	−0.002 (2)	−0.012 (2)
N1	0.042 (3)	0.045 (3)	0.045 (3)	−0.020 (2)	0.000 (2)	−0.017 (2)
C24	0.042 (3)	0.045 (3)	0.044 (3)	−0.013 (3)	0.004 (3)	−0.014 (3)
C23	0.048 (3)	0.058 (4)	0.045 (3)	−0.017 (3)	−0.001 (3)	−0.026 (3)
O4	0.062 (3)	0.091 (3)	0.068 (3)	−0.011 (2)	−0.024 (2)	−0.027 (2)
O2	0.074 (3)	0.077 (3)	0.062 (3)	−0.037 (2)	0.006 (2)	−0.013 (2)
C22	0.048 (3)	0.047 (3)	0.046 (3)	−0.012 (3)	−0.009 (3)	−0.008 (3)
C8	0.065 (4)	0.071 (4)	0.036 (3)	−0.041 (3)	0.005 (3)	−0.020 (3)
C21	0.040 (3)	0.053 (3)	0.038 (3)	−0.014 (3)	−0.001 (2)	−0.017 (3)
C7	0.047 (3)	0.062 (4)	0.038 (3)	−0.016 (3)	0.000 (3)	−0.016 (3)
C5	0.055 (4)	0.068 (4)	0.049 (3)	−0.022 (3)	−0.003 (3)	−0.030 (3)
C20	0.055 (4)	0.058 (4)	0.050 (3)	−0.022 (3)	−0.009 (3)	−0.022 (3)
O3	0.120 (4)	0.095 (3)	0.076 (3)	−0.006 (3)	−0.013 (3)	−0.056 (3)
C6	0.071 (4)	0.044 (3)	0.048 (3)	−0.021 (3)	−0.001 (3)	−0.021 (3)
C19	0.045 (3)	0.055 (3)	0.039 (3)	−0.012 (3)	−0.002 (3)	−0.018 (3)
C18	0.043 (3)	0.045 (3)	0.032 (3)	−0.013 (3)	0.000 (2)	−0.016 (2)
C3	0.072 (4)	0.060 (4)	0.039 (3)	−0.015 (3)	−0.005 (3)	−0.021 (3)
C17	0.034 (3)	0.068 (4)	0.068 (4)	−0.012 (3)	0.001 (3)	−0.013 (3)
C16	0.049 (4)	0.044 (3)	0.051 (3)	−0.014 (3)	0.006 (3)	−0.012 (3)
C2	0.064 (4)	0.054 (4)	0.042 (3)	−0.005 (3)	−0.017 (3)	−0.015 (3)
C4	0.063 (4)	0.053 (4)	0.042 (3)	−0.023 (3)	−0.008 (3)	−0.018 (3)
C15	0.050 (4)	0.065 (4)	0.046 (3)	−0.016 (3)	−0.021 (3)	−0.008 (3)
C14	0.076 (5)	0.036 (3)	0.056 (4)	−0.014 (3)	−0.003 (3)	−0.015 (3)
C13	0.065 (4)	0.049 (4)	0.053 (4)	−0.029 (3)	0.004 (3)	−0.009 (3)
C12	0.047 (4)	0.086 (5)	0.051 (3)	−0.032 (3)	−0.002 (3)	−0.035 (3)
C11	0.046 (4)	0.070 (4)	0.053 (4)	−0.007 (3)	0.010 (3)	−0.027 (3)
C10	0.052 (4)	0.067 (4)	0.060 (4)	−0.023 (3)	−0.015 (3)	−0.020 (3)
C1	0.063 (4)	0.078 (4)	0.033 (3)	−0.019 (3)	−0.005 (3)	−0.017 (3)
C9	0.053 (4)	0.053 (4)	0.058 (4)	−0.003 (3)	0.005 (3)	−0.018 (3)
O1	0.089 (3)	0.098 (3)	0.058 (3)	−0.009 (3)	−0.030 (2)	−0.022 (2)
O5	0.137 (4)	0.082 (3)	0.101 (3)	−0.030 (3)	−0.019 (3)	−0.059 (3)

### Geometric parameters (Å, °)

**Table:**

Fe1—C5	1.739 (6)	C8—C8^i^	1.552 (8)
Fe1—C4	1.745 (7)	C8—H8A	0.9700
Fe1—S2	2.2624 (14)	C8—H8B	0.9700
Fe1—P1	2.2670 (14)	C21—H21A	0.9300
Fe1—S1	2.2694 (13)	C7—H7A	0.9700
Fe2—C1	1.765 (6)	C7—H7B	0.9700
Fe2—C3	1.760 (6)	C5—O5	1.152 (5)
Fe2—C2	1.788 (6)	C20—C10	1.368 (8)
Fe2—S1	2.2723 (14)	C20—H20A	0.9300
Fe2—S2	2.2743 (14)	O3—C3	1.142 (5)
P1—C26	1.823 (5)	C6—H6A	0.9700
P1—C18	1.835 (4)	C6—H6B	0.9700
P1—C25	1.839 (4)	C19—C11	1.381 (6)
S2—C7	1.816 (6)	C19—H19A	0.9300
S1—C6	1.812 (4)	C18—C16	1.393 (6)
C26—C21	1.385 (6)	C17—C11	1.360 (7)
C26—C22	1.391 (6)	C17—C10	1.373 (7)
C25—C20	1.370 (7)	C17—H17A	0.9300
C25—C19	1.398 (6)	C16—C9	1.370 (6)
N1—C7	1.441 (5)	C16—H16A	0.9300
N1—C6	1.443 (5)	C15—C12	1.385 (6)
N1—C8	1.461 (5)	C15—H15A	0.9300
C24—C13	1.377 (6)	C14—C9	1.360 (6)
C24—C18	1.387 (5)	C14—C13	1.368 (6)
C24—H24A	0.9300	C14—H14A	0.9300
C23—C12	1.376 (6)	C13—H13A	0.9300
C23—C21	1.380 (6)	C12—H12A	0.9300
C23—H23A	0.9300	C11—H11A	0.9300
O4—C4	1.156 (5)	C10—H10A	0.9300
O2—C2	1.154 (7)	C1—O1	1.157 (6)
C22—C15	1.377 (6)	C9—H9A	0.9300
C22—H22A	0.9300		
			
C5—Fe1—C4	90.6 (2)	C23—C21—C26	121.6 (5)
C5—Fe1—S2	88.79 (16)	C23—C21—H21A	119.5
C4—Fe1—S2	158.94 (18)	C26—C21—H21A	119.5
C5—Fe1—P1	94.85 (16)	N1—C7—S2	114.2 (3)
C4—Fe1—P1	99.09 (16)	N1—C7—H7A	108.8
S2—Fe1—P1	102.17 (5)	S2—C7—H7A	108.8
C5—Fe1—S1	150.83 (16)	N1—C7—H7B	108.8
C4—Fe1—S1	86.21 (15)	S2—C7—H7B	108.8
S2—Fe1—S1	84.04 (4)	H7A—C7—H7B	107.7
P1—Fe1—S1	114.29 (5)	O5—C5—Fe1	177.9 (5)
C1—Fe2—C3	91.5 (2)	C10—C20—C25	121.5 (5)
C1—Fe2—C2	100.9 (2)	C10—C20—H20A	119.3
C3—Fe2—C2	99.1 (2)	C25—C20—H20A	119.3
C1—Fe2—S1	88.47 (17)	N1—C6—S1	115.5 (3)
C3—Fe2—S1	160.45 (16)	N1—C6—H6A	108.4
C2—Fe2—S1	100.14 (16)	S1—C6—H6A	108.4
C1—Fe2—S2	154.73 (16)	N1—C6—H6B	108.4
C3—Fe2—S2	88.13 (17)	S1—C6—H6B	108.4
C2—Fe2—S2	104.11 (16)	H6A—C6—H6B	107.5
S1—Fe2—S2	83.70 (5)	C11—C19—C25	120.0 (4)
C26—P1—C18	104.7 (2)	C11—C19—H19A	120.0
C26—P1—C25	103.0 (2)	C25—C19—H19A	120.0
C18—P1—C25	102.3 (2)	C24—C18—C16	117.2 (4)
C26—P1—Fe1	116.21 (13)	C24—C18—P1	123.6 (4)
C18—P1—Fe1	111.65 (14)	C16—C18—P1	119.2 (3)
C25—P1—Fe1	117.26 (15)	O3—C3—Fe2	179.8 (6)
C7—S2—Fe1	114.11 (16)	C11—C17—C10	120.5 (5)
C7—S2—Fe2	108.06 (16)	C11—C17—H17A	119.8
Fe1—S2—Fe2	67.70 (4)	C10—C17—H17A	119.8
C6—S1—Fe1	118.03 (16)	C9—C16—C18	121.1 (4)
C6—S1—Fe2	106.23 (17)	C9—C16—H16A	119.4
Fe1—S1—Fe2	67.62 (4)	C18—C16—H16A	119.4
C21—C26—C22	118.8 (4)	O2—C2—Fe2	178.3 (4)
C21—C26—P1	118.9 (4)	O4—C4—Fe1	176.3 (4)
C22—C26—P1	122.7 (4)	C22—C15—C12	120.7 (5)
C20—C25—C19	118.3 (4)	C22—C15—H15A	119.6
C20—C25—P1	119.3 (4)	C12—C15—H15A	119.6
C19—C25—P1	122.8 (3)	C9—C14—C13	119.4 (5)
C7—N1—C6	113.8 (4)	C9—C14—H14A	120.3
C7—N1—C8	115.0 (4)	C13—C14—H14A	120.3
C6—N1—C8	114.1 (4)	C14—C13—C24	120.5 (4)
C13—C24—C18	121.0 (4)	C14—C13—H13A	119.8
C13—C24—H24A	119.5	C24—C13—H13A	119.8
C18—C24—H24A	119.5	C23—C12—C15	119.5 (4)
C12—C23—C21	120.0 (5)	C23—C12—H12A	120.3
C12—C23—H23A	120.0	C15—C12—H12A	120.3
C21—C23—H23A	120.0	C17—C11—C19	119.9 (5)
C15—C22—C26	120.0 (4)	C17—C11—H11A	119.7
C15—C22—H22A	120.0	C19—C11—H11A	119.7
C26—C22—H22A	120.0	C17—C10—C20	119.2 (5)
N1—C8—C8^i^	115.5 (4)	C17—C10—H10A	120.4
N1—C8—H8A	108.4	C20—C10—H10A	120.4
C8^i^—C8—H8A	108.4	O1—C1—Fe2	178.3 (5)
N1—C8—H8B	108.4	C14—C9—C16	120.7 (5)
C8^i^—C8—H8B	108.4	C14—C9—H9A	119.6
H8A—C8—H8B	107.5	C16—C9—H9A	119.6
			
C5—Fe1—P1—C26	139.2 (2)	C25—P1—C26—C22	115.3 (4)
C4—Fe1—P1—C26	−129.4 (2)	Fe1—P1—C26—C22	−115.0 (3)
S2—Fe1—P1—C26	49.39 (18)	C26—P1—C25—C20	179.4 (4)
S1—Fe1—P1—C26	−39.52 (18)	C18—P1—C25—C20	−72.2 (4)
C5—Fe1—P1—C18	19.2 (2)	Fe1—P1—C25—C20	50.5 (4)
C4—Fe1—P1—C18	110.6 (2)	C26—P1—C25—C19	−4.5 (4)
S2—Fe1—P1—C18	−70.61 (16)	C18—P1—C25—C19	104.0 (4)
S1—Fe1—P1—C18	−159.52 (15)	Fe1—P1—C25—C19	−133.4 (3)
C5—Fe1—P1—C25	−98.6 (2)	C21—C26—C22—C15	−0.2 (6)
C4—Fe1—P1—C25	−7.2 (2)	P1—C26—C22—C15	177.6 (3)
S2—Fe1—P1—C25	171.60 (16)	C7—N1—C8—C8^i^	−71.4 (6)
S1—Fe1—P1—C25	82.94 (19)	C6—N1—C8—C8^i^	63.5 (7)
C5—Fe1—S2—C7	−160.8 (2)	C12—C23—C21—C26	0.4 (7)
C4—Fe1—S2—C7	110.8 (5)	C22—C26—C21—C23	0.0 (6)
P1—Fe1—S2—C7	−66.07 (17)	P1—C26—C21—C23	−177.8 (3)
S1—Fe1—S2—C7	47.55 (17)	C6—N1—C7—S2	71.8 (4)
C5—Fe1—S2—Fe2	98.66 (16)	C8—N1—C7—S2	−153.9 (3)
C4—Fe1—S2—Fe2	10.2 (4)	Fe1—S2—C7—N1	−69.5 (3)
P1—Fe1—S2—Fe2	−166.64 (4)	Fe2—S2—C7—N1	3.5 (4)
S1—Fe1—S2—Fe2	−53.08 (5)	C19—C25—C20—C10	0.1 (7)
C1—Fe2—S2—C7	−129.1 (4)	P1—C25—C20—C10	176.4 (4)
C3—Fe2—S2—C7	141.5 (2)	C7—N1—C6—S1	−64.6 (4)
C2—Fe2—S2—C7	42.6 (2)	C8—N1—C6—S1	160.1 (3)
S1—Fe2—S2—C7	−56.34 (16)	Fe1—S1—C6—N1	57.8 (4)
C1—Fe2—S2—Fe1	−19.8 (4)	Fe2—S1—C6—N1	−15.1 (4)
C3—Fe2—S2—Fe1	−109.24 (16)	C20—C25—C19—C11	−1.2 (7)
C2—Fe2—S2—Fe1	151.89 (16)	P1—C25—C19—C11	−177.4 (4)
S1—Fe2—S2—Fe1	52.97 (4)	C13—C24—C18—C16	−2.1 (7)
C5—Fe1—S1—C6	−120.6 (4)	C13—C24—C18—P1	175.2 (4)
C4—Fe1—S1—C6	155.0 (2)	C26—P1—C18—C24	104.2 (4)
S2—Fe1—S1—C6	−43.94 (19)	C25—P1—C18—C24	−2.7 (4)
P1—Fe1—S1—C6	56.75 (19)	Fe1—P1—C18—C24	−129.2 (3)
C5—Fe1—S1—Fe2	−23.5 (3)	C26—P1—C18—C16	−78.5 (4)
C4—Fe1—S1—Fe2	−107.90 (15)	C25—P1—C18—C16	174.6 (4)
S2—Fe1—S1—Fe2	53.14 (4)	Fe1—P1—C18—C16	48.0 (4)
P1—Fe1—S1—Fe2	153.83 (5)	C24—C18—C16—C9	1.6 (7)
C1—Fe2—S1—C6	−142.7 (2)	P1—C18—C16—C9	−175.8 (4)
C3—Fe2—S1—C6	127.2 (5)	C26—C22—C15—C12	−0.2 (7)
C2—Fe2—S1—C6	−41.9 (2)	C9—C14—C13—C24	−1.0 (8)
S2—Fe2—S1—C6	61.38 (16)	C18—C24—C13—C14	1.9 (8)
C1—Fe2—S1—Fe1	103.14 (16)	C21—C23—C12—C15	−0.8 (7)
C3—Fe2—S1—Fe1	13.1 (5)	C22—C15—C12—C23	0.7 (7)
C2—Fe2—S1—Fe1	−156.05 (16)	C10—C17—C11—C19	0.2 (8)
S2—Fe2—S1—Fe1	−52.84 (5)	C25—C19—C11—C17	1.1 (7)
C18—P1—C26—C21	−173.5 (3)	C11—C17—C10—C20	−1.3 (8)
C25—P1—C26—C21	−66.9 (4)	C25—C20—C10—C17	1.2 (7)
Fe1—P1—C26—C21	62.8 (4)	C13—C14—C9—C16	0.4 (8)
C18—P1—C26—C22	8.7 (4)	C18—C16—C9—C14	−0.8 (8)

Symmetry codes: (i) −*x*+1, −*y*+1, −*z*+1.

### Hydrogen-bond geometry (Å, °)

**Table:**

*D*—H···*A*	*D*—H	H···*A*	*D*···*A*	*D*—H···*A*
C20—H20A···O5^ii^	0.93	2.39	3.182 (7)	143

Symmetry codes: (ii) −*x*, −*y*, −*z*+1.
